# Efficacy and safety of Omega-3 polyunsaturated fatty acids in adjuvant treatments for colorectal cancer: A meta-analysis of randomized controlled trials

**DOI:** 10.3389/fphar.2023.1004465

**Published:** 2023-04-18

**Authors:** Haoshuang Liu, Jingfeng Chen, Weihao Shao, Su Yan, Suying Ding

**Affiliations:** ^1^ Health Management Center, The First Affiliated Hospital of Zhengzhou University, Zhengzhou, China; ^2^ College of Public Health, Zhengzhou University, Zhengzhou, China

**Keywords:** omega-3 polyunsaturated fatty acids, adjuvant treatments, colorectal cancer, efficacy and safety, meta-analysis

## Abstract

**Background:** Colorectal cancer (CRC) ranks third globally. There are many adverse reactions to treatments such as surgeries and post-surgical chemotherapy, which affect patients’ prognosis and reduce their life quality. Omega-3 polyunsaturated fatty acids (O3FAs) have become an essential part of immune nutrition due to their anti-inflammatory properties, which improve body immunity and have attracted widespread attention. A systematic review focused on the efficacy and safety of O3FAs for patients undergoing surgeries in combination with chemotherapy or a surgery alone is lacking.

**Objectives:** To evaluate the efficacy of O3FAs in the adjuvant treatment of CRC, a meta-analysis was conducted on patients with CRC who underwent surgeries in combination with chemotherapy or a surgery alone.

**Methods:** As of March 2023, publications have been obtained using search terms from digital databases such as PubMed, Web of Science, Embase and Cochrane Library. Only randomized clinical trials (RCTs) evaluating the efficacy and safety of O3FAs following adjuvant treatments for CRC were included in the meta-analysis. Key outcomes were tumor necrosis factor-alpha (TNF-α), C-reactive protein (CRP), interleukin-6 (IL-6), interleukin-1beta (IL-1β), albumin, body mass index (BMI), weight, the rate of infectious and non-infectious complications, the length of hospital stay (LOS), CRC mortality and life quality.

**Results:** After screening 1,080 studies, 19 RCTs (*n* = 1,556) with O3FAs in CRC were included, in all of which at least one efficacy or safety outcome was examined. Compared to the control group, the level of TNF-α (MD = −0.79, 95% CI: 1.51 to −0.07, *p* = 0.03) and IL-6 was reduced due to O3FA-enriched nutrition during the perioperative period (MD = −4.70, 95% CI: 6.59 to −2.80, *p* < 0.00001). It also reduces LOS (MD = 9.36, 95% CI: 2.16 to 16.57, *p* = 0.01). No significant differences were found in CRP, IL-1β, albumin, BMI, weight, the rate of infectious and non-infectious complications, CRC mortality or life quality. The inflammatory status of patients with CRC undergoing adjuvant therapies decreased after a total parenteral nutrition (TPN) O3FA supplementation (TNF-α, MD = −1.26, 95% CI: 2.25 to −0.27, *p* = 0.01, *I* ^*2*^ = 4%, *n* = 183 participants). The rate of infectious and non-infectious complications was reduced among patients with CRC undergoing adjuvant therapies after a parenteral nutrition (PN) O3FA supplementation (RR = 3.73, 95% CI: 1.52 to 9.17, *p* = 0.004, *I* ^*2*^ = 0%, *n* = 76 participants).

**Conclusion:** Our observations suggest that supplementation with O3FAs has little or no effect on patients with CRC undergoing adjuvant therapies and that a prolonged inflammatory state may be modified. To validate these findings, well-designed, large-scale, randomized and controlled studies on homogeneous patient populations are expected.

## Introduction

In the Global Cancer Report (GLOBOCAN) 2020, colorectal cancer (CRC) ranked as the third most common cancer worldwide and the second leading cause of cancer-related deaths worldwide (Sung et al., 2021). Chemotherapy is a standard adjuvant treatment for CRC (Benson et al., 2018), for which the most typical treatments are surgical removal and chemotherapy. Patients’ nutritional and inflammatory status may be altered via these treatments, which may affect their life quality and prognosis. Malnourished patients with cancers have poorer clinical outcomes, higher rates of complications and longer hospital stays. High-inflammation patients with cancers have a shorter overall, disease-free and progression-free survival (Zhou et al., 2021).

Experiments have shown that eicosapentaenoic acid (EPA) and docosahexaenoic acid (DHA), two of the most common long-chain O3FAs produced from marine sources, exhibit anti-inflammatory and anti-CRC effect (Calder, 2010; Cockbain et al., 2012; Calder, 2015). The body cannot produce necessary fatty acids, known as O3FAs, although they can be found in fish fats and vegetable oils in high concentrations. Both DHA and EPA are critical O3FAs that have received the most research attention (Xie and Chang, 2016; Calder, 2017). O3FAs have the potential to play a role in multiple stages of CRC management, starting with the primary CRC prevention and continuing to the “tertiary” prevention stage following CRC treatments and advanced metastatic diseases.

According to the findings of a study conducted by Mocellin and his colleagues, O3FAs raised the level of plasma albumin and prealbumin in patients with stomach cancer (Mocellin et al., 2018). Ma’s research showed that O3FAs lowered C-reactive protein (CRP) level and shortened the duration of systemic inflammatory response syndrome (Ma et al., 2016). Based on an earlier meta-analysis of all surgical patients, O3FAs may have improved clinical outcomes, including infection rates and hospital stays (Calder, 2010). On the other hand, Lam’s research has shown that O3FAs have no substantial effect on the nutritional improvement or inflammatory regulations of cancerous patients (Lam et al., 2021). Considering that the results and conclusions of these studies were not completely consistent due to limited sample size, different study designs, and potential bias, we conducted a meta-analysis of all relevant randomized controlled trials (RCTs), focusing on the effects of O3FAs on nutritional status, inflammation, and immune function of patients after CRC chemotherapy, providing a theoretical basis for the standardized clinical application of O3FAs in CRC patients. Moreover, the results of several recent RCTs are controversial. The aim of this systematic review is therefore to assess the potential role of O3FA in the outcomes of postoperative CRC patients.

## Methods

Our systematic review was conducted according to the guidelines in Preferred Reporting Items for Systematic Reviews and Meta-Analyses (PRISMA) (Page et al., 2021, Supplementary Table S6).

### Search strategy

A systematic literature search was conducted based on databases such as PubMed, Web of Science, Embase, and Cochrane from inception to March 2023. Search results are further restricted to RCTs. The search terms were Omega-3, polyunsaturated fatty acid and colorectal neoplasms associated with these terms. Separate search strategies (PubMed, Web of Science, Embase, Cochrane) were designed for each database, which are included in Supplement 1 (Supplementary Tables S1–S4).

We also identified ongoing and recently-completed trials by searching ClinicalTrials.gov. No attempt was made to search for unpublished studies. In addition, only papers published in English were included. Reviews, laboratory studies, case-control studies, cohort studies, case reports, abstracts, letters and editorials that did not meet the outcomes of interventions in this review were excluded, as were articles that lacked sufficient information or relevant results. If multiple published data points pointed to the same patient cohort as the one under investigation, only the most current or comprehensive studies were selected.

### Eligibility criteria and selection of studies

Four separate viewers (Haoshuang Liu, Jingfeng Chen, Weihao Shao, and Su Yan) reviewed citation titles, abstracts and the entire texts in duplicate. To be included in this review, the design had to be an RCT. The inclusion criteria are presented in Table 1. Only complete publications (not conference abstracts) and articles published in English shall be submitted for consideration. Results were compared and disputes were resolved based on consensus.

**TABLE 1:** Inclusion and exclusion criteria in the review.

	Inclusion criteria	Exclusion criteria
Population	Adult Undergo surgery in combination with chemotherapy or surgery alone	Children Animal data Healthy volunteers
Study type	Randomized controlled trials (RCTs)	Reviews/editorials/case reports non-RCTs/Published as an abstract
Intervention	All routes of administration were eligible (i.e., oral, PN, TPN or EN), alone or in combination with other parenteral or enteral feeding regimes	Arginin Glutamine RNA
Outcomes of interest	At least one of the Following outcomes:	
	Weight	
	Rates of infectious and non-infectious complications	
	Length of hospital stay (LOS)	
	CRC mortality	
	Tumor necrosis factor-α (TNF-α)	
	C-reactive protein (CRP)	
	Albumin	
	BMI (Body Mass Index)	
	IL-6 (interleu-6)	
	IL-1β(interleukin-1beta)	
	Quality of life	

Abbreviations: PN, parenteral nutrition; TPN, total parenteral nutrition; EN, enteral nutrition.

### Data extraction

Based on publications available, we collected information on the outcomes of each study included. Results of interest, including mean and standard deviation (mean ± SD) of each group after interventions, were then retrieved for each study included. As is previously stated, the resulting data was extracted. Additional study characteristics such as the first author, study publication year, study follow-up duration, sample size of interventions and the control group, study design as well as participant characteristics were also collected for study inclusion. We made a list of the most significant criteria and then checked the quality of the studies we included, all of which were RCTs.

### Assessment of study strength and quality

The Cochrane risk of bias tools (Cumpston et al., 2022) was used to independently review the quality of included studies of three authors (Haoshuang Liu, Weihao Shao, and SuYan), two (Jingfeng Chen and Weihao Shao) of whom discussed the quality of those studies and agreed. If they could not agree, a third author (Suying Ding) was brought in. To evaluate the study methodologies, we used the Cochrane collaboration bias risk tool. Through RCTs, the following traits are measures based on the scale:

1) Randomly-generated sequences (selection bias), 2) allocation concealment (selection bias), 3) the use of a blinding technique for respondents and administrators selected (performance bias), 4) the use of a blinding technique to evaluate findings (detection bias), 5) insufficient outcome data (attrition bias), 6) selective reporting (reporting bias), and 7) other biases.

The number of components for which a high risk of biases (ROBs) was likely to be present in trials was used to group trials into three ROB categories: high-risk (five or more), moderate-risk (3 or 4) and low-risk (two or less).

### Meta-analytic methods

Two reviewers (Su Yan and Suying Ding) worked separately to extract data from specially-constructed Microsoft Excel spreadsheets. The ROB of each study was assessed as “high,” “unclear” or “low.” Methods, participants, interventions and outcome measures as well as other relevant characteristics and outcomes of the studies were aggregated by reviewers. Any difference among reviewers was addressed through discussion and, where feasible, based on pooling additional information from study investigators. In patients with CRC, outcome measures such as tumor necrosis factor-alpha (TNF-α), CRP, interleukin-6 (IL-6), interleukin-1beta (IL-1β), albumin, body mass index (BMI), weight, the rate of infectious and non-infectious complications, the length of hospital stay (LOS), CRC mortality and life quality were compared between groups.

Meta-analyses were performed through Review Manager 5.4.1. For each study included, mean and variance data on continuous variables was imported into Manager 5.4.1. Where possible, the mean ± SD of post-intervention values of both intervention and control group was used. For studies reported using the median and interquartile range, mean and SD were calculated using a method described by Wan et al. (2014). For dichotomous outcomes, the number of participants per group (intervention and control group) in the events was meta-analyzed to produce a relative risk (RR) with a 95% confidence interval (CI).

O3FAs can be delivered via either parenteral or enteral routes. Therefore, controlling internal heterogeneity in study design is a challenge for systematic review researchers. We developed a strategy including *I* ^*2*^ tests, nutritional support routes and EPA doses for subgroup analyses on O3FA preparations, as well as the timing of pre- or post-operative administration, so as to reduce heterogeneity. Subgroup analyses were also performed according to the TNM stage. We were unable to subgroup based on changes in the omega-3/omega-6 ratio (as is anticipated) since such information was rarely given. Subsequently, *I* ^*2*^ statistical tests were used to determine the degree of statistical heterogeneity, and either random-effect or fixed-effect models were selected for further analyses. RR or mean difference (MD) with a 95% CI was used to show the combined effect size (MD = absolute difference between the mean of both groups, defined as the difference between the mean of the treatment and control group calculated using the same scale). Publication biases were assessed using the Review Manager 5.4.1 package, based on a Begg and Egger test. Statistical significance was defined as *p* < 0.05.

## Results

### Search results and study quality

**FIGURE 1:**
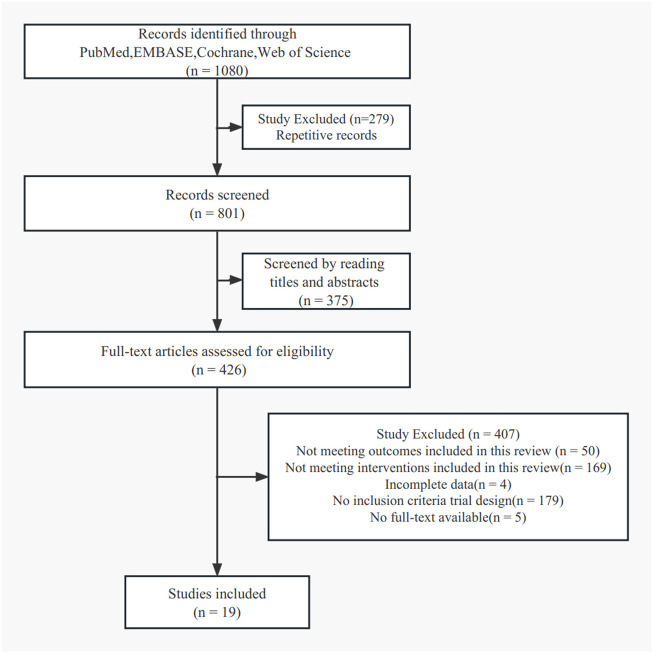
Flow diagram of the selection statergy.

**FIGURE 2:**
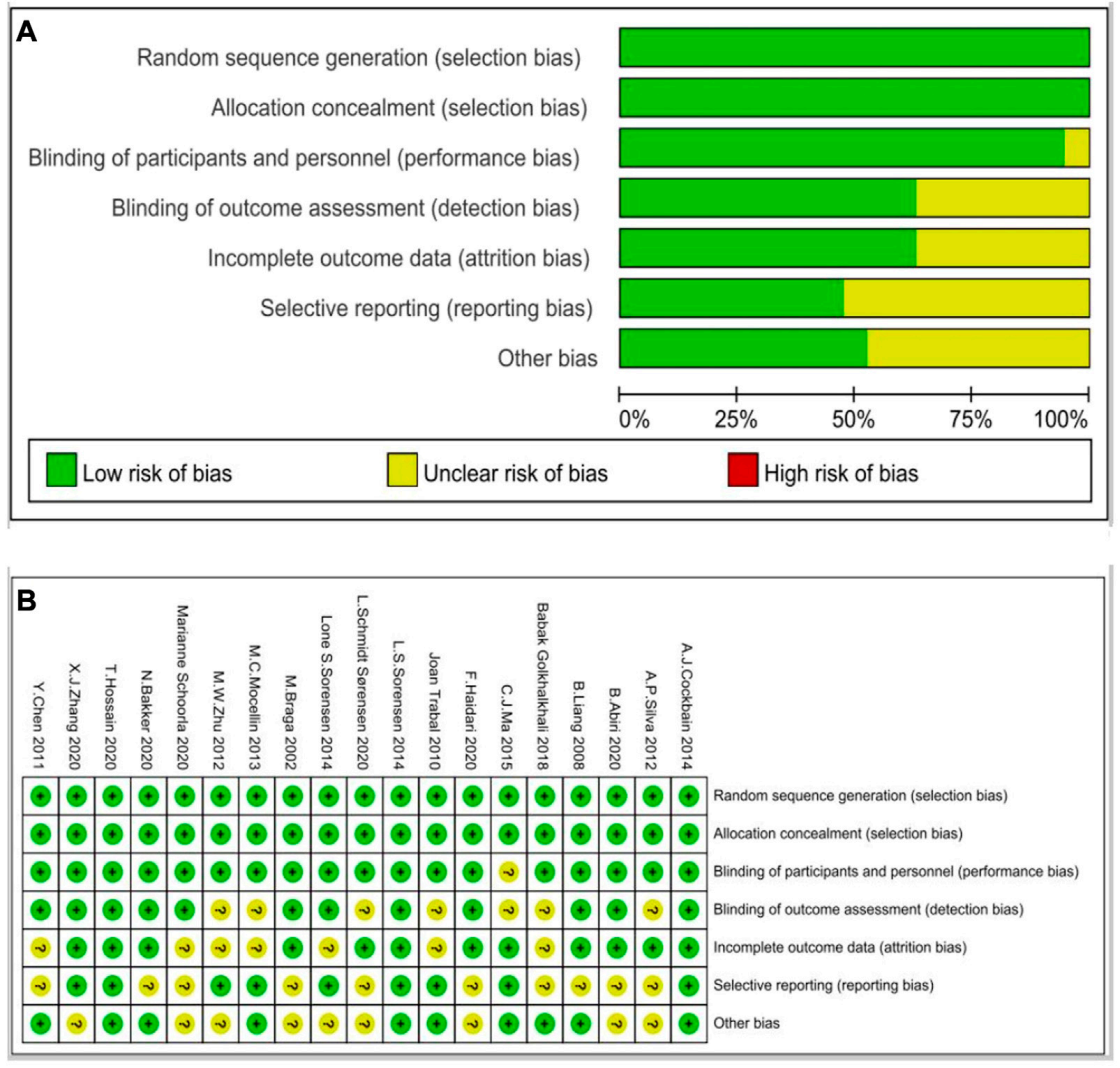
Risk of bias graph of RCTs **(A)**; Risk of bias summary of RCTs **(B)**.

A search of electronic literatures resulted in 1,080 eligible articles. By reviewing the titles and abstracts only, we narrowed the number of potentially-relevant articles to 426. This meta-analysis consists of 19 eligible papers in total. Two investigators (Jingfeng Chen and Weihao Shao) completed all the procedures separately. RCTs are of a moderate to high quality. Figure 1 shows the retrieval and selection flowchart. Figures 2A, B show the graphical results of methodological quality based on the extracted information.

### Characteristics of studies included

The basic characteristics of the selected studies are summarized in Table 2. We included 19 RCTs (Braga et al., 2002; Liang et al., 2008; Trabal et al., 2010; Chen et al., 2011; Silva et al., 2012; Zhu et al., 2012; Mocellin et al., 2013; Sorensen et al., 2014a; Cockbain et al., 2014; Ma et al., 2015; Golkhalkhali et al., 2018; Bakker et al., 2020a; Haidari et al., 2020a; 2020b; Hossain et al., 2020; Sørensen et al., 2020; Zhang et al., 2020) measuring at least one of our efficacy or safety outcomes. Studies on 11 (Mocellin et al., 2013) and 179 (Zhang et al., 2020) participants included the sample size. The majority of studies included patients undergoing surgeries (*n* = 11) (Braga et al., 2002; Liang et al., 2008; Chen et al., 2011; Zhu et al., 2012 Sorensen et al., 2014a; 2014b; Ma et al., 2015; Bakker et al., 2020a; 2020b; Hossain et al., 2020; Sørensen et al., 2020), with the remainder conducted on patients receiving chemotherapy (*n* = 2) (Trabal et al., 2010; Zhang et al., 2020) or that in combination with surgeries (*n* = 6) (Silva et al., 2012; Mocellin et al., 2013; Cockbain et al., 2014; Golkhalkhali et al., 2018; Haidari et al., 2020b). The duration of interventions ranged from 2 (Bakker et al., 2020a) to 84 days (Trabal et al., 2010). O3FA-enriched oral capsules were used in 12 studies **(** Zhang et al., 2020; Haidari et al., 2020a; 2020b; Hossain et al., 2020; Sørensen et al., 2020; Golkhalkhali et al., 2018; Sorensen et al., 2014a; Cockbain et al., 2014; Mocellin et al., 2013; Silva et al., 2012; Trabal et al., 2010), total parenteral nutrition (TPN) was used in four studies (Liang et al., 2008; Chen et al., 2011; Zhu et al., 2012; Ma et al., 2015), PN was used in two of them (Bakker et al., 2020a; 2020b), and EN was used in the rest (Braga et al., 2002). The prescribed dose of O3FA ranged from 0.108 g/d (Haidari et al., 2020b; Zhang et al., 2020) to 4.00 g/d (Sørensen et al., 2020; Sorensen et al., 2014a; 2014b). EPA doses below 1.80 g/d were used in 15 (Zhang et al., 2020; Haidari et al., 2020a; 2020b; Bakker et al., 2020a; Golkhalkhali et al., 2018; Ma et al., 2015; Cockbain et al., 2014; Mocellin et al., 2013; Zhu et al., 2012; Silva et al., 2012; Chen et al., 2010; Trabal et al., 2010; Liang et al., 2008; Braga et al., 2002) studies, while doses above 1.80 g/d were used in four (Hossain et al., 2020; Sørensen et al., 2020; Sorensen et al., 2014a) studies.

**TABLE 2:** Characteristics of included studies.

Id	Mean age (mean ± SD)	Total/male/female	Population	LOS (day)	Dosage (g/d)	Administration route	Frequency	Control (n)	O3FA (n)	Parameter	Staging
Zhang et al. (2020)	T:NA	T:90/47/43	chemotherapy	30	EPA:0.064	PO	tid	89	90	⑨	TNM: III, Ⅳ
	C:NA	C:89/46/43									
Haidari et al. (2020a)	T:56.75 ± 10.60	T:20/12/8	chemotherapy + surgery	56	EPA:0.108	PO	qd	20	20	⑤⑥⑩	TNM: II, III
	C:59.90 ± 8.75	C:20/8/12									
Haidari et al. (2020b)	T:56.75 ± 10.60	T:20/12/8	chemotherapy + surgery	57	EPA:0.108	PO	qd	20	20	①②③⑤⑩⑪	TNM: II, III
	C:59.90 ± 8.76	C:20/8/12									
Bakker et al. (2020a)	T: 65 [64,72]	T:17/12/5	surgery	2	EPA:0.135	PN	qd	18	17	③⑤⑦⑧	TNM:I, II, III
	C: 68 [63,73]	C:18/12/6									
Marianne Schoorla 2020	T:65.00 (12.00)	T:18/13/5	surgery	2	EPA:0.163	PN	qd	23	18	⑦	TNM:I, II, III
	C:69.00 (11.00)	C:23/15/8									
Hossain et al. (2020)	T: 69.03 (7.95)	T:32/24/8	surgery	26	EPA:1.000	PO	tid	29	32	⑦⑧	Duke:A, B, C
	C: 67.42 (6.92)	C:29/18/11									
Sørensen et al. (2020)	T: 68.30 (11.30)	T:65/27/38	surgery	14	EPA:2.000	PO	bid	60	65	④⑦⑧	UICC:I, II, III, Ⅳ
	C: 70.60 (10.10)	C:60/30/30									
Golkhalkhali et al. (2018)	T:NA	T:70	chemotherapy + surgery	56	EPA:0.700	PO	qd	70	70	②③⑤⑨⑩⑪	NA
	C:NA	C:70									
Ma et al. (2015)	T: 61.55 ± 9.78	T:51/29/22	surgery	8	EPA:0.900	TPN	qd	48	51	②③⑤⑦⑩	UICC:I, II, III, Ⅳ
	C:62.85 ± 10.12	C:48/27/21									
Sorensen et al. (2014a)	T: 69.00 (11.00)	T:74/44/30	surgery	14	EPA:2.000	PO	bid	74	74	④⑦⑧	TNM:I, II, III, Ⅳ
	C: 71.00 (10.00)	C:74/36/38									
Sorensen et al. (2014b)	T: 69.00 (11.00)	T:74/44/30	surgery	7	EPA:2.000	PO	bid	74	74	④	TNM:I, II, III, Ⅳ
	C: 71.00 (10.00)	C:74/36/38									
Cockbain et al. (2014)	T: 68 (44–82)	T:43/26/17	chemotherapy + surgery	30 (12–65)	EPA:0.600	PO	bid	45	43	④⑦	Duke:A, B, C, D
	C: 71 (35–87)	C:45/35/10									
Mocellin et al. (2013)	T: 55.20 (7.70)	T:6/3/3	chemotherapy + surgery	63	EPA:0.360	PO	qd	5	6	①②③⑥⑩⑪	TNM:III, Ⅳ
	C: 53.60 (12.90)	C:5/3/2									
Zhu et al. (2012)	T: 69.80 ± 10.50	T:29/16/13	surgery	7	EPA:0.600	TPN	qd	28	29	⑤⑦⑧⑩	Duke:B, C
	C: 70.80 ± 6.40	C:28/17/11									
Silva etal. (2012)	T: 50.10 (8.20)	T:11/3/8	chemotherapy + surgery	63	EPA:0.180	PO	qd	12	11	①②③⑤⑥⑩⑪	TNM:I, II, III, Ⅳ
	C: 54.30 (9.30)	C:12/3/9									
Chen et al. (2011)	T: NA	T:102/49/53	surgery	NA	EPA:0.180	TPN	NA	65	102	④⑦⑧	NA
	C: NA	C:65									
Trabal et al. (2010)	T: 61.50 ± 15.80	T:6/4/2	chemotherapy	84x	EPA:1.600	PO	NA	7	6	⑦⑨⑪	TNM: Ⅳ
	C: 68.20 ± 15.60	C:7/5/2									
Liang et al. (2008)	T: 55.80 ± 10.10	T:20/10/10	surgery	7	EPA:0.600	TPN	qd	21	20	⑤⑦⑧⑩	TNM:I, II, III
	C: 59.19 ± 10.61	C:21/15/6									
Braga et al. (2002)	T: 60.50 ± 11.50	T:50/28/22	surgery	T:9.80 ± 3.10 C:12.2 ± 3.90	EPA:1.152	EN	qd	50	50	④⑦⑧	NA
	C: 62.20 ± 10.40	C:50/29/21									

①Albumin; ②BMI; ③C-reactive protein (CRP); ④CRC, mortality; ⑤IL-6 (interleukin-6); ⑥IL-1β; ⑦rates of infectious and non-infectious complications; ⑧Length of hospital stay (LOS); ⑨Quality of life; ⑩Tumor necrosis factor-α (TNF-α); ⑪Weight. LOS, length of hospital stay; O3FA, omega-3 polyunsaturated fatty acids; NA, not available; T, treatment; C, control; PO, per oral; PN, parenteral nutrition; TPN, total parenteral nutrition; EN, enteral nutritiopn.

### The effect of O3FA supplementation on the postoperative level of inflammatory factors

#### The effect of O3FA supplementation on TNF-α

**FIGURE 3:**
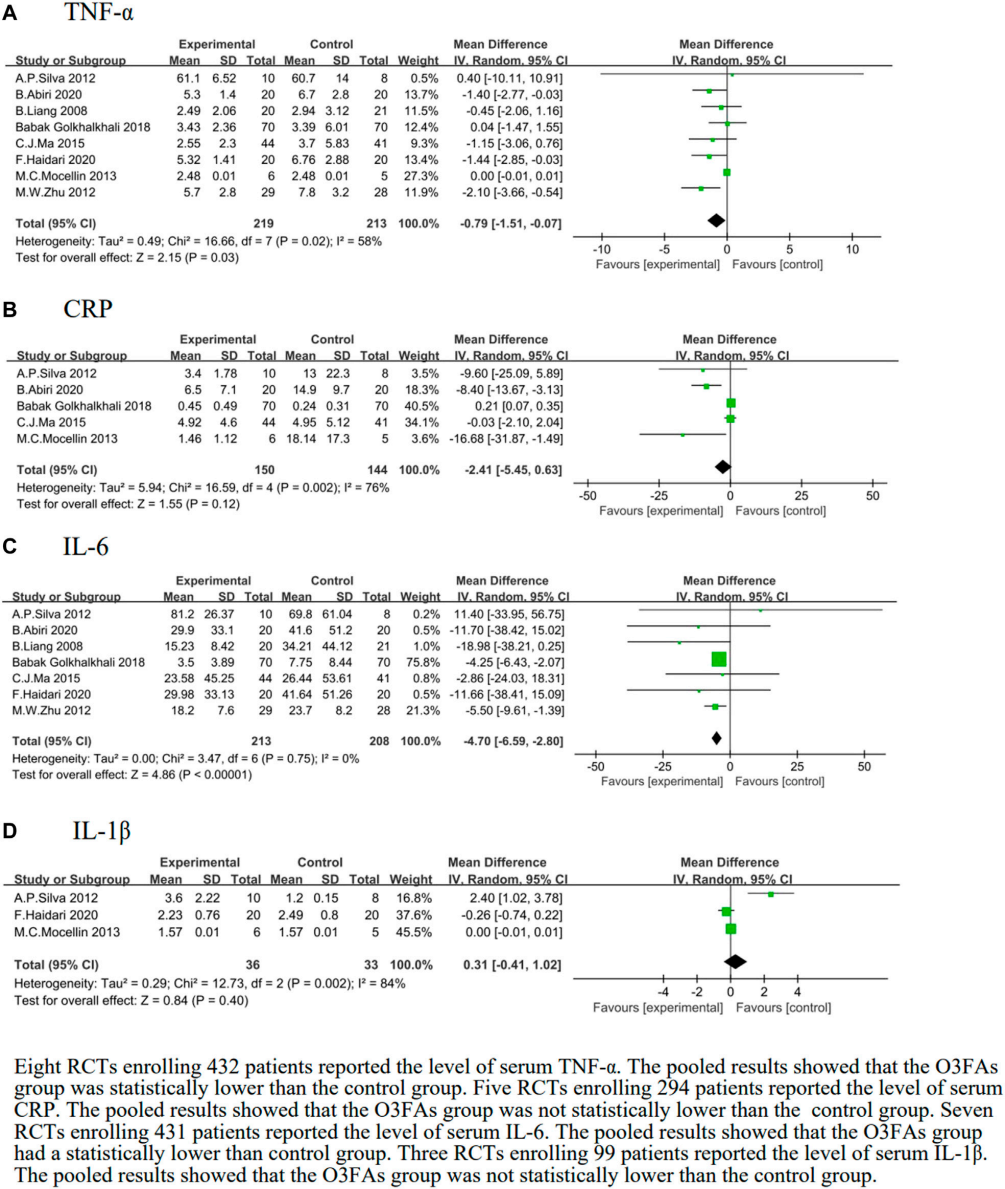
Effect of O3FAs on postoperative level of inflammatory factor **(A)**: TNF-α, **(B)**: CRP, **(C)**IL-6, **(D)**: IL-1β.

We identified 8 eligible trials (Liang et al., 2008; Silva et al., 2012; Zhu et al., 2012; Mocellin et al., 2013; Ma et al., 2015; Golkhalkhali et al., 2018; Haidari et al., 2020b), which included 432 patients, and the TNF-α level following postoperative O3FA supplementation was investigated versus the control group. The results of the heterogeneity test were *p* = 0.02 and *I* ^2^ = 58%. The meta-analysis revealed that the TNF-α level effectively decreased with O3FAs compared to the control group (MD = −0.79, 95% CI: −1.51 to −0.07, *p* = 0.03, Figure 3A).

After subgroup analyses on different routes of administration, there was a positive trend of TNF-α when O3FAs were used in TPN (MD = −1.26, 95% CI: −2.25 to −0.27, *p* = 0.01, *I* ^*2*^ = 4%, *n* = 183 participants, Supplementary Table S5).

#### The effect of O3FA supplementation on CRP

We identified five eligible trials (Silva et al., 2012; Mocellin et al., 2013; Ma et al., 2015 Golkhalkhali et al., 2018; Haibari et al., 2020b), which included 294 patients, and investigated peripheral blood CRP levels following the postoperative O3FA supplementation compared to the control group. The results of the heterogeneity test were *p* = 0.002 and *I* ^*2*^ = 76%. The forest plot indicated that there was no statistical significance in the CRP of the O3FAs group and the control group (MD = −2.41, 95% CI: −5.45 to 0.63, *p* = 0.12, Figure 3B).

#### The effect of O3FA supplementation on IL-6

We identified seven eligible trials (Liang et al., 2008; Silva et al., 2012; Zhu et al., 2012; Ma et al., 2015; Golkhalkhali et al., 2018; Haidari et al., 2020a; 2020b), which included 421 patients, and investigated IL-6 levels following the postoperative O3FA supplementation versus the control group. The heterogeneity test results were *p* = 0.75 and *I* ^*2*^ = 0%. The pooled results of the O3FAs group were statistically lower than those of the control group (MD = −4.70, 95% CI: −6.59 to −2.80, *p* < 0.00001, Figure 3C).

#### The effect of O3FA supplementation on IL-1β

We identified three eligible trials (Silva et al., 2012; Mocellin et al., 2013; Haidari et al., 2020b), which included 69 patients, and investigated IL-1β levels following the postoperative O3FA supplementation versus the control group. The heterogeneity test results were *p* = 0.002 and *I* ^*2*^ = 84%. The forest plot indicated that there was no statistical significance in the IL-1β level of the O3FAs group and the control group (MD = 0.31, 95% CI: −0.41 to 1.02, *p* = 0.40, Figure 3D).

### The effect of O3FAs on postoperative nutritional status

#### The effect of O3FA supplementation on albumin

**FIGURE 4:**
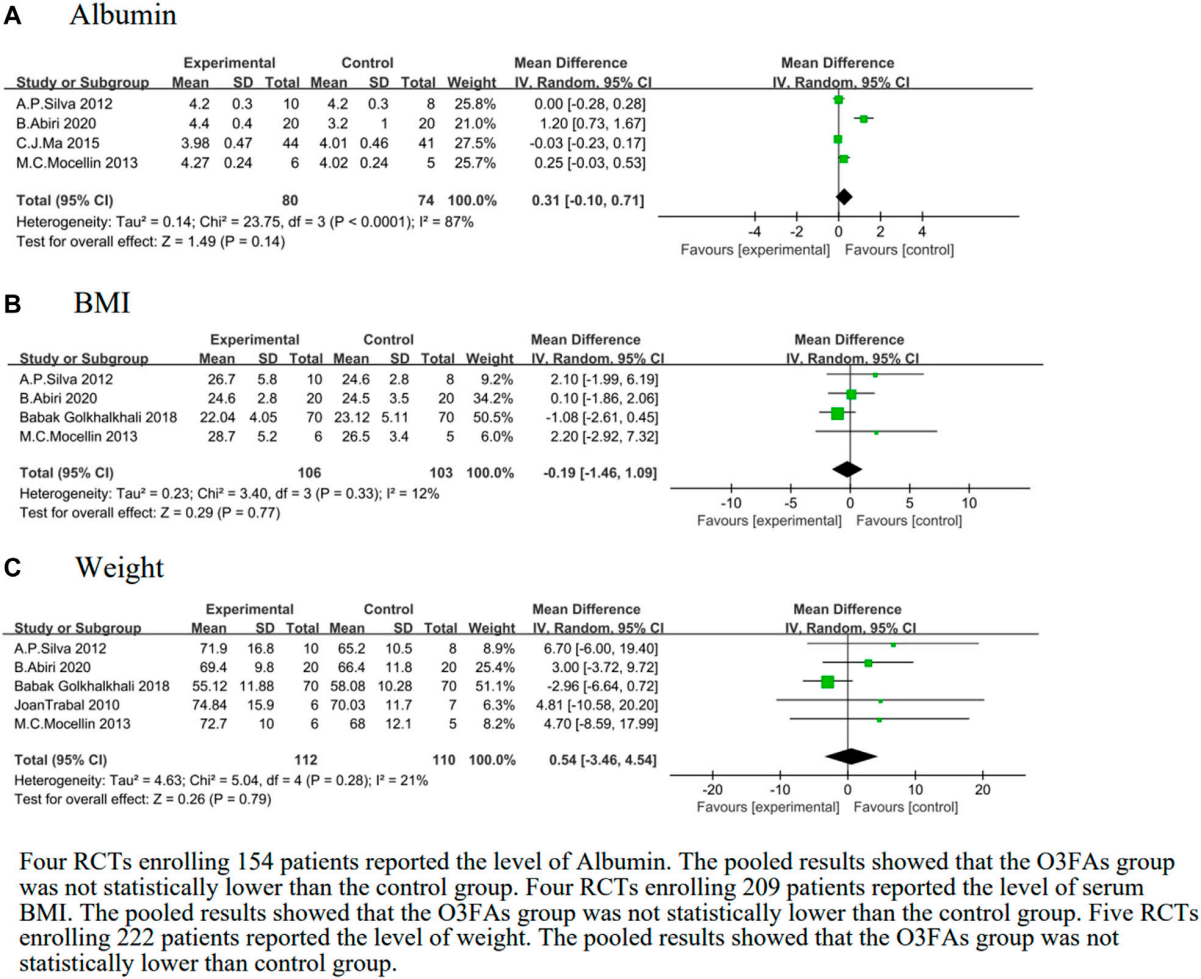
Effect of O3FAs on postoperative nutritional status **(A)**: Alubumin, **(B)**: BMI, **(C)**: Weight.

Four studies (Silva et al., 2012; Mocellin et al., 2013; Ma et al., 2015; Haidari et al., 2020b) were pooled to estimate the effect size of O3FAs on the albumin of O3FA-supplemented and the control group. The heterogeneity test results were *p* < 0.0001 and *I* ^*2*^ = 87%. The O3FA supplementation did not affect albumin compared to the control group (MD = 0.31, 95% CI: −0.10 to 0.71, *p* = 0.14, *n* = 154 participants, Figure 4A).

#### The effect of O3FA supplementation on BMI

We identified four eligible trials (Silva et al., 2012 Mocellin et al., 2013 Golkhalkhali et al., 2018; Haidari et al., 2020b), which included 209 patients, and investigated the associations between O3FAs and BMI. The heterogeneity test results were *p* = 0.33 and *I* ^*2*^ = 12%. The O3FA supplementation did not affect BMI compared to the control group (MD = −0.19, 95% CI: −1.46 to 1.09, *p* = 0.77, Figure 4B).

#### The effect of O3FA supplementation on weight

We identified five eligible trials (Trabal et al., 2010; Silva et al., 2012; Mocellin et al., 2013; Golkhalkhali et al., 2018; Haidari et al., 2020b), which included 222 patients, and investigated the associations between O3FAs and weight. Results of the heterogeneity test were *p* = 0.28 and *I* ^*2*^ = 21%. The forest plot showed no statistically-significant weight difference between the O3FAs group and the control group (MD = 0.54, 95% CI: −3.46 to 4.54, *p* = 0.79, Figure 4C).

#### The effect of O3FA supplementation on the rate of infectious and non-infectious complications

**FIGURE 5:**
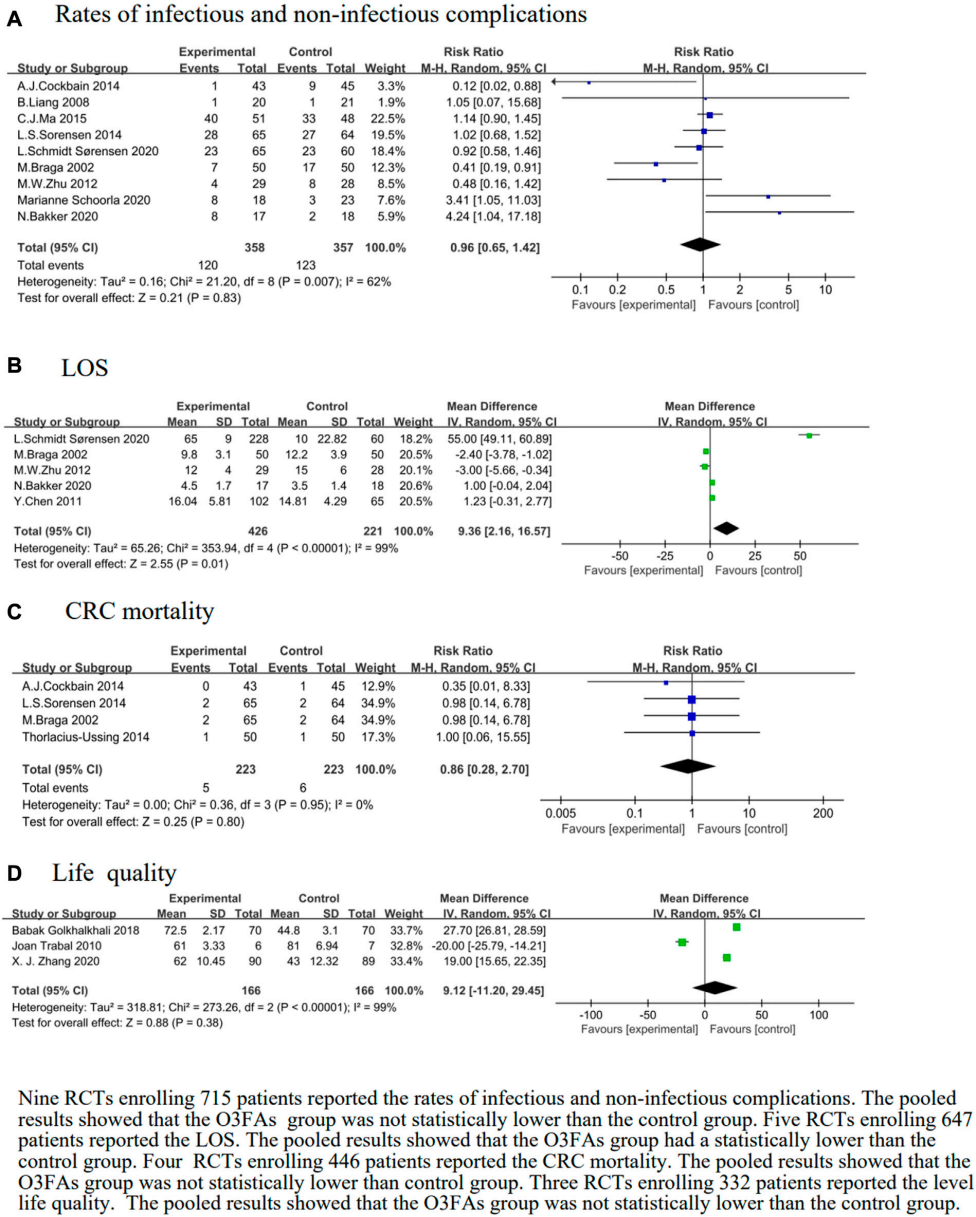
Effect of O3FAs onrates of infectious and non-infectious complications **(A)**,LOS **(B)**, CRC mortality **(C)**, life quality **(D)**.

The rate of infectious and non-infectious complications was reported in nine trials (Braga et al., 2002; Liang et al., 2008; Zhu et al., 2012; Sorensen et al., 2014b; Cockbain et al., 2014; Ma et al., 2015; Bakker et al., 2020a; 2020b; Sørensen et al., 2020). The heterogeneity test results were *p* = 0.007 and *I* ^*2*^ = 62%. The forest plot indicated that there was no statistical significance in the rate of infectious and non-infectious complications of the O3FAs group or the control group (RR = 0.96, 95% CI: 0.65 to 1.42, *p* = 0.83, *n* = 715 participants, Figure 5A).

Subgroup analyses showed no significant differences stratified by duration, but lower rates of infectious and non-infectious complications were shown in the O3FAs group than in the control group with PN (RR = 3.73, 95% CI: 1.52 to 9.17, *p* = 0.004, *I* ^*2*^ = 0%, *n* = 76 participants, Supplementary Table S5).

#### The effect of O3FA supplementation on the LOS

We identified five eligible trials (Braga et al., 2002; Chen et al., 2011; Zhu et al., 2012; Bakker et al., 2020a; Sørensen et al., 2020). The heterogeneity test results were *p* < 0.00001 and *I* ^*2*^ = 99%. The pooled results of the O3FAs group was statistically lower than those of the control group (MD = 9.36, 95% CI: 2.16 to 16.57, *p* = 0.01, *n* = 647 participants, Figure 5B).

#### The effect of O3FA supplementation on CRC mortality

We identified four eligible trials (Braga et al., 2002; Sorensen et al., 2014a; Cockbain et al., 2014), which included 446 patients, and investigated the associations between O3FAs and CRC mortality. The heterogeneity test results were *p =* 0.95 and *I* ^*2*^ = 0%. The forest plot indicated that there was no statistical significance in the CRC mortality level of the O3FAs group and the control group (RR = 0.86, 95% CI: 0.28 to 2.70, *p* = 0.80, Figure 5C).

#### The effect of O3FA supplementation on life quality

We identified three eligible trials (Trabal et al., 2010; Golkhalkhali et al., 2018; Zhang et al., 2020), which included 332 patients, and investigated the associations between O3FAs and life quality. The heterogeneity test results were *p <* 0.00001 and *I* ^*2*^ = 99%. The forest plot indicated that there was no statistical significance in the life quality of the O3FAs group or the control group (RR = 9.12, 95% CI: −11.20 to 29.45, *p* = 0.38, Figure 5D).

### Subgroup analysis

For the other subgroups [dosages (EPA >1.80 g/d or <1.80 g/d) and TNM staging (TNM staging II, III or IV)], no significant differences were observed between the O3FAs and the control group (Supplementary Table S5).

### Assessment of reporting biases

Using funnel plots, Egger and Begg tested publication biases. In terms of accuracy, the funnel plot is not as good as Egger’s test or Begg’s test, and Begg’s test is not as sensitive as Egger’s test. When the three results do not agree, they first discard the funnel plot. When Egger’s and Begg’s test results are opposite, Egger’s test result is used as the result, and the trim-and-fill method will be used to adjust publication biases through a meta-analysis. When three or more studies are included for one outcome, Egger’s test is used to assess publication biases (Egger et al., 1997; Sterne and Davey Smith, 2001). Egger’s test showed that the *p* values were >0.05, indicating no publication bias.

## Discussion

Multiple immunosuppressive nutrients in nutritional formulations for patients with massive tumors after surgeries are recommend in ESPEN guidelines for improved outcomes. As early as possible (within 5–7 days) before surgeries, nutritional supplementation should begin and continue during the postoperative period (Weimann et al., 2021). Through our meta-analysis, 19 RCTs were evaluated to assess the effect and safety of O3FA nutritional support on inflammatory cytokine levels, nutritional status, infectious and non-infectious complications, LOS, CRC mortality as well as life quality of patients with CRC. The main outcome of this study was that the serum TNF-α and IL-6 level of patients with CRC undergoing chemotherapy or surgeries alone was effectively reduced with a nutritional support for O3FAs. Patients’ LOS was reduced with O3FAs by enhancing body immune function and reducing body inflammatory response, but they did not modulate CRP, IL-1β, albumin, BMI, weight, the rate of infectious and non-infectious complications, CRC mortality or life quality. According to different durations, different routes of administration, dosing of O3FAs and TNM staging, we conducted subgroup analyses and found that O3FAs improved the TNF-α level of patients treated with TPN, as well as infectious and non-infectious complications of patients treated with PN.

O3FAs can inhibit the activation of epidermal growth factor receptors (EGFRs), thereby inhibiting the phosphorylation of growth-factor-receptor-binding protein 2 (Grb2), which play a role in inflammation inhibition (D'Angelo, et al., 2020; Calder, 2017). O3FAs can also inhibit inflammation by inhibiting toll-related receptors, downregulating the NF-κB signaling pathway and reducing inflammatory gene expression (Volpato and Hull, 2018). Supplementing O3FAs benefits cancerous patients because it lowers the level of inflammatory cytokines, including IL-6 and TNF-α (Mayer and Seeger, 2008; Singer et al., 2008). IL-6 is a type of inflammatory cytokine that is mostly produced by immune cells (such as T cells), endothelial cells and macrophages, which inhibits the stress response. It is effective in modulating the immune system and fighting against infections. O3FAs, according to substantial published research, can reduce the level of postoperative IL-6 and TNF-α of cancerous patients (Goodla and Xue, 2022; Lu et al., 2022). Both cytokines caused CD4^+^ T cells to develop into various types of T-helper (Th) cells, and the TNF-α/IL-6 level mirrored the Th1–Th2 cell balance to some extent. Th1 cells are responsible for cellular immune responses, which play a key role in infection and tumor defense, while Th2 cells are responsible for humoral immune response. Patients with CRC exhibited a Th1–Th2 imbalance that shifted to Th2, which, exacerbated by operating stress, was found to be strongly associated with postoperative infections (Tabata et al., 1999; Tatsumi et al., 2003). Its effect on CD4^+^ and CD8^+^ T cell balance is also unknown due to the lack of studies. These findings support the hypothesis that a supplementation of O3FAs induces an anti-inflammatory or attenuated inflammatory response (Mayer et al., 2003; Barbosa et al., 2010a; Im, 2012; Al-Leswas et al., 2018).

Among several inflammatory agents, IL-6 ranks high in both importance and sensitivity. During surgeries, IL-6 stimulates the livers to produce acute-phase CRP, which boosts the phagocytic activity of neutrophils and macrophages. The level of IL-6 and CRP released in the body may reflect the its stress state. O3FAs have the potential to reduce CRP production, limit IL-6 release, lower the level of inflammatory response and improve the body immune function. Numerous previous studies have shown that O3FAs can reduce the level of IL-6 and TNF-α of cancerous patients following surgeries (Bernabe-Garcia et al., 2016; Guo et al., 2022; Kavyani et al., 2022), which is consistent with the findings of our meta-analysis.

Albumin is a key component of plasma total protein content. Albumin and BMI are also crucial indicators for nutritional assessments. Studies have shown that O3FAs can improve the nutritional status of patients (Feijó et al., 2019; Cheng et al., 2021), which are also recommended as fatty acid supplements in the nutritional treatment of various diseases (Stavrinou et al., 2020; Elagizi et al., 2021). However, a study involving 2,157 adults aged 70 or older showed no effect of taking O3FAs on patients’ nutritional status (Bischoff-Ferrari et al., 2020). It was found in our meta-analysis that O3FAs did not increase the albumin or BMI level. Due to the limited and varied number of trials included, there is a theoretical bias in our interpretation of the results of the two nutritional indicators studied.

Body weight was reported in five studies, and no significant difference was found between the O3FA supplementation group and the control group in our meta-analysis. Our findings were in contrast to a recent systematic review (Wang et al., 2021), which could be explained by the differences among studies in the current systematic review, including cancer types, different routes of administration, anti-cancer therapies and tolerance to anti-cancer therapies. The current systematic review includes studies on patients with CRC and any type of cancer treatment.

In our study, O3FAs did not affect the rate of infectious and non-infectious complications. Compared to Gao’s and Chen’s study, where the incidence of postoperative infection complications and LOS (Calder, 2010; Gao et al., 2020) was reduced with the supplementation of O3FAs, Gln, Arg or nucleotides, nutritional intervention methods might be used to partially explain these discrepancies. Studies have also shown that O3FAs do not significantly reduce the LOS level of patients with postoperative colon cancer receiving a PN therapy (Lee et al., 2023). However, a recent network meta-analysis indicated that LOS was shortened with parenteral O3FA supplementation (Pradelli et al., 2023), which was consistent with our meta-analysis results.

CRC mortality was reported in four studies, and no significant difference was found between the O3FA-supplement group and the control group in our meta-analysis. This finding contradicted previous studies (Barbosa et al., 2010b; Hall et al., 2015), which could be explained by differences in the study populations of the current systematic review, including disease type and treatment tolerance. The more homogeneous population and treatment in these studies may have led to different findings regarding the efficacy of O3FA supplementation.

Quality of life is an important patient outcome reported in cancer care. Inconsistent effect of O3FA supplements on life quality has been found in several previous systematic reviews. In one meta-analysis (Lam et al., 2021), including 31 studies, it was concluded that O3FA treatment did not improve life quality. A meta-analysis (de van der Schueren et al., 2018) revealed that O3FAs brought significant life quality benefits after supplementation. While both meta-analyses indicated consistent findings, which were based on small, limited compliance studies. Further studies are needed to determine the effect of O3FA supplementation on life quality.

Evidence to date suggests that the provision of O3FAs through capsules, oral nutrition supplements or enteral or parental formulas may help regulate the inflammatory environment in a number of medical conditions, which is linked in many cases to improved functions, clinical courses and outcomes (Gorjao et al., 2019; Klassen et al., 2020). Because dysregulated inflammation is a component of many acute and chronic diseases, the potential applications of DHA and EPA are broad in terms of prevention and treatment. There is positive evidence that O3FAs are safe and cost-effective therapies with the potential to benefit multiple patient outcomes. There is no doubt that the dose of DHA and EPA used is an essential factor, but it is not the only explanation for the inconsistencies. Additional considerations include the timing and duration of DHA and EPA supply, the ratio of EPA to DHA, baseline EPA and DHA status, the intake of other nutrients including omega-6 fatty acids, B vitamins and antioxidants, clinical status as well as medication use. More well-designed intervention studies are needed to address the relevance of these different variables to properly identify the effect of DHA and EPA on specific target patient populations. Such studies may lead to a more personalized approach to the provision of DHA and EPA for the maximum clinical benefits. A focus on personalized approaches and the knowledge of a patient’s specific nutritional and medical needs will be important in determining the paths of the optimal use of O3FAs, which should take into account interactions between genetics and nutrients as well as among nutrients themselves. Overall, the full body of evidence supports the use of DHA and EPA in a wide range of medical conditions. Additionally, high-quality studies based on the experience of existing studies will strengthen the evidence base needed to inform future guidelines.

Some limitations of this study merit a consideration. First of all, in most cases, participants’ adherence to the study treatment and food consumption was not tracked or reported, which might have affected the accuracy of the results. Secondly, instead of using a variation from the baseline value, we used the final value in our meta-analysis, which did not account for differences in the baseline of trials and might have affected pooled results. No follow-up questions were asked about dietary patterns, additional medical conditions or medications after surgeries, which should be considered as a restriction. Thirdly, raw data for several studies was missing, leading to their elimination from quantitative analyses. Different doses of O3FAs and varying amounts of EPA and DHA used in exploratory products were significant constraints for evaluating efficacy. Because dose-response studies on O3FAs are sparse, it is difficult to determine the effect of these changes, and we are unable to recommend an appropriate dose range. Despite the above limitations, our study suggests that there is no data in the current literatures that raises any concern about the safety of using O3FAs for patients with CRC.

In addition, alterations in inflammatory measures of TNF-α and IL-6 for patients with CRC confirm the immunological effect of O3FAs. In future investigations on the effect of O3FAs on chemotherapy and advanced CRC, gut microbiome changes should be examined. Despite promising preclinical data on combination therapies and intriguing preliminary clinical data, the efficacy of O3FA supplementation in combination with surgeries or chemotherapy for CRC has not been definitively tested clinically.

## Conclusion

In conclusion, our study indicated that O3FAs were safe and effective in lowering TNF-α and IL-6 level and shortening LOS for patients with CRC undergoing adjuvant therapies. However, analyses of these trials failed to show improvements in CRP, IL-1β, albumin, BMI, weight, infectious and non-infectious complication rates or life quality. In the future, exploration with the help of metabolomics, proteomics, microbiome, and other methods, based on the results of more clinical studies, can provide more references for the clinical application and promotion of nutritional therapies and standardized nutritional preparations in CRC patients.

## Data Availability

The original contributions presented in the study are included in the article/Supplementary Material, further inquiries can be directed to the corresponding author.
