# Supplementary material for: Efficacy and safety of Omega-3 polyunsaturated fatty acids in adjuvant treatments for colorectal cancer: A meta-analysis of randomized controlled trials
Source: Front Pharmacol. 2023 Apr 18;14:1004465. doi: 10.3389/fphar.2023.1004465 (PMC10151497; doi:10.3389/fphar.2023.1004465)
Supplement: Supplementary file 2 [file DataSheet4.PDF]

Table S6 Cochrane risk of bias

Study ID	Selection Bias		Performance Bias	Detection Bias	Attrition Bias	Reporting Bias	Other Bias
	Random sequence generation	Allocation concealment	Blinding of participants and personnel	Blinding of outcome assessment	Incomplete outcome data	Selective reporting	Other Bias
X.J.Zhang 2020	low	low	low	low	low	low	unclear
F.Haidari 2020	low	low	low	low	low	unclear	unclear
B.Abiri 2020	low	low	low	low	low	unclear	unclear
N.Bakker 2020	low	low	low	low	low	unclear	low
Marianne Schoorla 2020	low	low	low	low	unclear	unclear	unclear
T.Hossain 2020	low	low	low	low	low	low	low
L.Schmidt Sørensen 2020	low	low	low	unclear	low	unclear	unclear
Babak Golkhalkhali 2018	low	low	low	unclear	unclear	unclear	low
C.J.Ma 2015	low	low	unclear	unclear	low	low	low
L.S.Sorensen 2014	low	low	low	low	low	low	low
Lone S.Sorensen 2014	low	low	low	low	unclear	low	unclear
A.J.Cockbain 2014	low	low	low	low	low	low	low
M.C.Mocellin 2013	low	low	low	unclear	unclear	low	low
M.W.Zhu 2012	low	low	low	unclear	unclear	low	unclear
A.P.Silva 2012	low	low	low	unclear	low	unclear	unclear
Y.Chen 2011	low	low	low	unclear	unclear	unclear	low
Joan Trabal 2010	low	low	low	unclear	unclear	low	low
B.Liang 2008	low	low	low	low	low	unclear	low
M.Braga 2002	low	low	low	low	low	unclear	unclear
