# Supplementary material for: Efficacy and safety of Omega-3 polyunsaturated fatty acids in adjuvant treatments for colorectal cancer: A meta-analysis of randomized controlled trials
Source: Front Pharmacol. 2023 Apr 18;14:1004465. doi: 10.3389/fphar.2023.1004465 (PMC10151497; doi:10.3389/fphar.2023.1004465)
Supplement: Supplementary file 3 [file DataSheet3.pdf]

Table S5 Effects of O3FA on adjuvant treatments for colorectal cancer based on subgroup analysis

	Subgrouped by	No.of trials	No. of participants	Meta-analysis			Heterogeneity	
				MD	95%CI	*p*-Value	I^2(%)	Cochrane Q-test (*p*-Value)
TNF-α	Routes of administration							
	PO	5	249	-0.52	-1.31,0.26	0.19	50	0.09
	TPN	3	183	-1.26	-2.25,-0.27	0.01	4	0.35
	Timing of pre- or post- operative administration							
	Before the surgery	4	305	-1.01	-1.77,-0.26	0.00	0	0.47
	After the surgery	4	127	-0.64	-1.77,0.50	0.27	59	0.06
CRP	Routes of administration							
	PO	4	209	-6.55	-14.16,1.05	0.09	82	0.00
	TPN	1	85	-0.03	-2.10,2.04	0.98	NA	NA
	Timing of pre- or post- operative administration							

	Before the surgery	3	265	-1.32	-4.01,1.37	0.34	81	0.00
	After the surgery	2	29	-18.32	-26.11,-10.52	0.00	0	0.81
IL-6	Routes of administration							
	PO	4	238	-4.31	-6.47,-2.15	0.00	0	0.79
	TPN	3	183	-5.98	-9.92,-2.03	0.00	0	0.39
	Timing of pre- or post- operative administration							
	Before the surgery	4	305	-4.33	-6.48,-2.18	0.00	0	0.90
	After the surgery	3	116	-6.99	-14.99,1.01	0.09	16	0.31
IL-1β	Routes of administration							
	PO	NA	NA	NA	NA	NA	NA	NA
	TPN	NA	NA	NA	NA	NA	NA	NA
	Timing of pre- or post- operative administration							
	Before the surgery	1	40	-0.26	-0.74,0.22	0.29	NA	NA
	After the surgery	2	29	1.10	-1.25,3.44	0.36	91	0.00
Albumin	Routes of administration							
	PO	3	69	0.45	-0.13,1.04	0.13	89	0.00

	TPN	1	85	-0.03	-0.23,0.17	0.77	NA	NA
	Timing of pre- or post- operative administration							
	Before the surgery	2	125	0.57	-0.64,1.77	0.36	95	0.00
	After the surgery	2	29	0.12	-0.12,0.37	0.32	34	0.22
BMI	Routes of administration							
	PO	NA	NA	NA	NA	NA	NA	NA
	TPN	NA	NA	NA	NA	NA	NA	NA
	Timing of pre- or post- operative administration							
	Before the surgery	2	180	-0.64	-1.84,0.57	0.30	0	0.35
	After the surgery	2	29	2.14	-1.05,5.33	0.19	0	0.98
Weight	Routes of administration							
	PO	NA	NA	NA	NA	NA	NA	NA
	TPN	NA	NA	NA	NA	NA	NA	NA
	Timing of pre- or post- operative administration							
	Before the surgery	2	180	-0.67	-6.35,5.01	0.82	57	0.13

	After the surgery	3	40	5.41	-2.15,12.96	0.16	0	0.97
LOS	Routes of administration							
	PO	1	125	-1.00	-9.00,7.00	0.81	NA	NA
	TPN	2	224	-0.74	-4.88,3.40	0.73	86	0.00
	PN	1	35	1.00	-0.04,2.04	0.06	NA	NA
	EN	1	100	-2.40	-3.78,-1.02	0.00	NA	NA
	Timing of pre- or post- operative administration							
	Before the surgery	3	260	-0.70	-3.69,2.29	0.65	87	0.00
	After the surgery	2	224	-0.74	-4.88,3.40	0.73	86	0.00
Quality of life	Routes of administration							
	PO	NA	NA	NA	NA	NA	NA	NA
	TPN	NA	NA	NA	NA	NA	NA	NA
	Timing of pre- or post- operative administration							
	Before the surgery	1	140	27.70	26.81,28.59	0.00	NA	NA
	After the surgery	2	192	-0.43	-38.64,37.79	0.98	99	0.00
Dosage	EPA <1.80g/d	15	1074	0.98	0.88,1.09	0.68	0	0.86
	EPA >1.80g/d	4	482	1.13	0.96,1.34	0.14	5	0.37
TNM staging	TNM staging II	7	285	0.93	0.74,1.17	0.54	0	0.89

TNM staging III	9	475	1.06	0.88,1.28	0.56	0	0.96
TNM staging IV	4	310	0.98	0.81,1.17	0.79	0	0.99

	Subgrouped by	No.of trials	No. of participants	Meta-analysis			Heterogeneity	
				RR	95%CI	*p*-Value	I^2(%)	Cochrane Q-test (*p*-Value)
Rates of infectious and non-infectious complications	Routes of administration							
	PO	3	342	0.85	0.49,1.46	0.55	57	0.10
	TPN	3	87	0.95	0.53,1.68	0.85	27	0.25
	PN	2	76	3.73	1.52,9.17	0.00	0	0.82
	EN	1	24	0.41	0.19,0.91	0.03	NA	NA
	Timing of pre- or post- operative administration							

	Before the surgery	7	617	1.02	0.67,1.56	0.92	68	0.00
	After the surgery	2	98	0.54	0.20,1.47	0.23	0	0.60
CRC mortality	Routes of administration							
	PO	3	446	0.86	0.28,2.70	0.80	0	0.95
	EN	1	100	1.00	0.06,15.55	1.00	NA	NA
	Timing of pre- or post- operative administration							
	Before the surgery	NA	NA	NA	NA	NA	NA	NA
	After the surgery	NA	NA	NA	NA	NA	NA	NA
Therapy	Chemotherapy	2	192	1.00	0.82,1.22	1.00	0	0.69
	Surgery	11	1022	1.07	0.96,1.19	0.23	31	0.15
	Surgery combined with chemotherapy	6	342	0.99	0.85,1.15	0.88	0	1

O3FA,omega-3 polyunsaturated fatty acid; tumor necrosis factor-alpha,TNF-α; C-reactive protein,CRP; interleukin-6,IL-6; interleukin-1beta,IL-1β; body mass index,BMI; length of hospital stay,LOS; total parenteral nutrition, TPN; parenteral nutrition,PN; peros,PO; enteral nutrition,EN; eicosapentaenoic acid,EPA.
