# Supplementary material for: Efficacy and safety of Omega-3 polyunsaturated fatty acids in adjuvant treatments for colorectal cancer: A meta-analysis of randomized controlled trials
Source: Front Pharmacol. 2023 Apr 18;14:1004465. doi: 10.3389/fphar.2023.1004465 (PMC10151497; doi:10.3389/fphar.2023.1004465)
Supplement: Supplementary file 4 [file DataSheet1.PDF]

Table S1	Search strategy of PubMed
Search	Query
#1	“Fatty Acids, Omega-3” [Mesh Terms]
#2	((((((((((((((((((((((((((((((Fatty Acids, Omega-3[Mesh Terms]) OR (Omega-3 Fatty Acid[Title/Abstract])) OR (Acid, Omega-3 Fatty[Title/Abstract])) OR (Fatty Acid, Omega-3[Title/Abstract])) OR (Omega 3 Fatty Acid[Title/Abstract])) OR (Omega-3 Fatty Acids[Title/Abstract])) OR (n-3 Oil[Title/Abstract])) OR (Oil, n-3[Title/Abstract])) OR (n 3 Oil[Title/Abstract])) OR (n3 Oil[Title/Abstract])) OR (Oil, n3[Title/Abstract])) OR (n-3 Fatty Acids[Title/Abstract])) OR (n 3 Fatty Acids[Title/Abstract])) OR (Omega 3 Fatty Acids[Title/Abstract])) OR (n-3 PUFA[Title/Abstract])) OR (PUFA, n-3[Title/Abstract])) OR (n 3 PUFA[Title/Abstract])) OR (n3 Fatty Acid[Title/Abstract])) OR (Fatty Acid, n3[Title/Abstract])) OR (n3 PUFA[Title/Abstract])) OR (PUFA, n3[Title/Abstract])) OR (n3 Polyunsaturated Fatty Acid[Title/Abstract])) OR (n3 Oils[Title/Abstract])) OR (n-3 Oils[Title/Abstract])) OR (n 3 Oils[Title/Abstract])) OR (N-3 Fatty Acid[Title/Abstract])) OR (Acid, N-3 Fatty[Title/Abstract])) OR (Fatty Acid,

	N-3[Title/Abstract])) OR (N 3 Fatty Acid[Title/Abstract])) OR (n-3 Polyunsaturated Fatty Acid[Title/Abstract])) OR (n 3 Polyunsaturated Fatty Acid[Title/Abstract])
#3	"Fatty Acids, Unsaturated" [Mesh Terms]
#4	(((((((((((Fatty Acids, Unsaturated[MeSH Terms]) OR (Acids, Unsaturated Fatty[Title/Abstract])) OR (Unsaturated Fatty Acids[Title/Abstract])) OR (Unsaturated Fatty Acid[Title/Abstract])) OR (Acid, Unsaturated Fatty[Title/Abstract])) OR (Fatty Acid, Unsaturated[Title/Abstract])) OR (Polyunsaturated Fatty Acids[Title/Abstract])) OR (Acids, Polyunsaturated Fatty[Title/Abstract])) OR (Fatty Acids, Polyunsaturated[Title/Abstract])) OR (Polyunsaturated Fatty Acid[Title/Abstract])) OR (Acid, Polyunsaturated Fatty[Title/Abstract])) OR (Fatty Acid, Polyunsaturated[Title/Abstract])
#5	"Colorectal Neoplasms" [Mesh Terms]
#6	((((((((((((((Colorectal Neoplasms[MeSH Terms]) OR (Colorectal Neoplasm[Title/Abstract])) OR (Neoplasm, Colorectal[Title/Abstract])) OR (Neoplasms, Colorectal[Title/Abstract])) OR (Colorectal Tumors[Title/Abstract])) OR (Colorectal

	Tumor[Title/Abstract])) OR (Tumor, Colorectal[Title/Abstract])) OR (Tumors, Colorectal[Title/Abstract])) OR (Colorectal Cancer[Title/Abstract])) OR (Cancer, Colorectal[Title/Abstract])) OR (Cancers, Colorectal[Title/Abstract])) OR (Colorectal Cancers[Title/Abstract])) OR (Colorectal Carcinoma[Title/Abstract])) OR (Carcinoma, Colorectal[Title/Abstract])) OR (Carcinomas, Colorectal[Title/Abstract])) OR (Colorectal Carcinomas[Title/Abstract])
#7	#2 AND #4 AND #6

Table S2	Search strategy of Web of Science
Search	Query
#1	"Colorectal Neoplasms" [Topic]
#2	((((((((((((((TS=(Colorectal Neoplasms)) OR TS=(Colorectal Neoplasm)) OR TS=(Neoplasm, Colorectal)) OR TS=(Neoplasms, Colorectal)) OR TS=(Colorectal Tumors)) OR TS=(Colorectal Tumor)) OR TS=(Tumor, Colorectal)) OR TS=(Tumors, Colorectal)) OR TS=(Colorectal Cancer)) OR

	TS=(Cancer, Colorectal)) OR TS=(Cancers, Colorectal)) OR TS=(Colorectal Cancers)) OR TS=(Colorectal Carcinoma)) OR TS=(Carcinoma, Colorectal)) OR TS=(Carcinomas, Colorectal)) OR TS=(Colorectal Carcinomas)
#3	"Fatty Acids, Omega-3" [Topic]
#4	((((((((((((((((((((((((((((((TS=(Fatty Acids, Omega-3)) OR TS=(Omega-3 Fatty Acid)) OR TS=(Acid, Omega-3 Fatty)) OR TS=(Fatty Acid, Omega-3)) OR TS=(Omega 3 Fatty Acid)) OR TS=(Omega-3 Fatty Acids)) OR TS=(n-3 Oil)) OR TS=(Oil, n-3)) OR TS=(n 3 Oil)) OR TS=(n3 Oil)) OR TS=(Oil, n3)) OR TS=(n-3 Fatty Acids)) OR TS=(n 3 Fatty Acids)) OR TS=(Omega 3 Fatty Acids)) OR TS=(n-3 PUFA)) OR TS=(PUFA, n-3)) OR TS=(n 3 PUFA)) OR TS=(n3 Fatty Acid)) OR TS=(Fatty Acid, n3)) OR TS=(n3 PUFA)) OR TS=(PUFA, n3)) OR TS=(n3 Polyunsaturated Fatty Acid)) OR TS=(n3 Oils)) OR TS=(n-3 Oils)) OR TS=(n 3 Oils)) OR TS=(N-3 Fatty Acid)) OR TS=(Acid, N-3 Fatty)) OR TS=(Fatty Acid, N-3)) OR TS=(N 3 Fatty Acid)) OR TS=(n-3 Polyunsaturated Fatty Acid)) OR TS=(n 3 Polyunsaturated Fatty Acid)
#5	"Fatty Acids, Unsaturated" [Topic]
#6	(((((((((((TS=(Fatty Acids, Unsaturated)) OR TS=(Acids, Unsaturated Fatty)) OR TS=(Unsaturated Fatty Acids)) OR

	TS=(Unsaturated Fatty Acid)) OR TS=(Acid, Unsaturated Fatty)) OR TS=(Fatty Acid, Unsaturated)) OR TS=(Polyunsaturated Fatty Acids)) OR TS=(Acids, Polyunsaturated Fatty)) OR TS=(Fatty Acids, Polyunsaturated)) OR TS=(Polyunsaturated Fatty Acid)) OR TS=(Acid, Polyunsaturated Fatty)) OR TS=(Fatty Acid, Polyunsaturated)
#7	#2 AND #4 AND #6

Table S3	Search strategy of Embase
Search	Query
#1	Mesh descriptor: [Colorectal Neoplasms] explode all trees
#2	(Colorectal Neoplasm or Neoplasm, Colorectal or Neoplasms, Colorectal or Colorectal Tumors or Colorectal Tumor or Tumor, Colorectal or Tumors, Colorectal or Colorectal Cancer or Cancer, Colorectal or Cancers, Colorectal or Colorectal Cancers or Colorectal Carcinoma or Carcinoma, Colorectal or Carcinomas, Colorectal or Colorectal Carcinomas): ti,ab,kw
#3	#1 OR #2
#4	Mesh descriptor: [Fatty Acids, Omega-3] explode all trees
#5	(Omega-3 Fatty Acids or n-3 Oil or Oil, n-3 or n 3 Oil or n3 Oil or Oil, n3 or n-3 Fatty Acids or n 3 Fatty Acids or Omega 3 Fatty Acids or n-3 PUFA or PUFA, n-3 or n 3 PUFA or n3 Fatty

	Acid or Fatty Acid, n3 or n3 PUFA or PUFA, n3 or n3 Polyunsaturated Fatty Acid or n3 Oils or n-3 Oils or n 3 Oils or N-3 Fatty Acid or Acid, N-3 Fatty or Fatty Acid, N-3 or N 3 Fatty Acid or n-3 Polyunsaturated Fatty Acid or n 3 Polyunsaturated Fatty Acid): ti,ab,kw
#6	#4 OR #5
#7	Mesh descriptor: [Fatty Acids, Unsaturated] explode all trees
#8	(Acids, Unsaturated Fatty or Unsaturated Fatty Acids or Unsaturated Fatty Acid or Acid, Unsaturated Fatty or Fatty Acid, Unsaturated or Polyunsaturated Fatty Acids or Acids, Polyunsaturated Fatty or Fatty Acids, Polyunsaturated or Polyunsaturated Fatty Acid or Acid, Polyunsaturated Fatty or Fatty Acid, Polyunsaturated):ti,ab,kw
#9	#7 OR #8
#10	#3 AND #6 AND #9

Table S4	Search strategy of Cochrane
Search	Query
#1	'omega 3 fatty acid'/exp OR 'bilantin omega':ti,ab,kw OR 'conchol 36':ti,ab,kw OR 'eicosa e':ti,ab,kw OR eicosapen:ti,ab,kw OR epaisdin:ti,ab,kw

	OR epanova:ti,ab,kw OR 'fatty acids, omega 3':ti,ab,kw OR 'fatty acids, omega-3':ti,ab,kw OR 'n 3 fatty acid':ti,ab,kw OR 'n 3 polyunsaturated fatty acid':ti,ab,kw OR 'omega 3':ti,ab,kw OR 'omega 3 carboxylic acid':ti,ab,kw OR 'omega 3 carboxylic acids':ti,ab,kw OR 'omega 3 feingold':ti,ab,kw OR 'omega 3 plus':ti,ab,kw OR 'omega 3 polyunsaturated fatty acid':ti,ab,kw OR 'omega forte':ti,ab,kw OR 'omega-3-carboxylic acids':ti,ab,kw OR 'omega3 polyunsaturated fatty acid':ti,ab,kw OR 'sakana; sanhelios omega 3':ti,ab,kw
#2	'polyunsaturated fatty acid'/exp OR 'fatty acid polyunsaturation':ti,ab,kw OR 'fatty acid, polyunsaturated':ti,ab,kw OR 'poly unsaturated fatty acid':ti,ab,kw OR 'polyunsaturated fat':ti,ab,kw
#3	'colorectal neoplasms':ti,ab,kw OR 'colorectal neoplasm':ti,ab,kw OR 'neoplasm, colorectal':ti,ab,kw OR 'neoplasms, colorectal':ti,ab,kw OR 'colorectal tumors':ti,ab,kw OR 'colorectal tumor':ti,ab,kw OR 'tumor, colorectal':ti,ab,kw OR 'tumors, colorectal':ti,ab,kw OR 'colorectal cancer':ti,ab,kw OR 'cancer, colorectal':ti,ab,kw OR 'cancers, colorectal':ti,ab,kw OR 'colorectal cancers':ti,ab,kw OR 'colorectal carcinoma':ti,ab,kw

	OR 'carcinoma, colorectal':ti,ab,kw OR 'carcinomas, colorectal':ti,ab,kw OR 'colorectal carcinomas':ti,ab,kw
#4	#1 AND #2 AND #3
